# The Correlation Between the Presence of *BRAF^V600E^* and *TERT* Promoter Mutation and the Response to Treatment with Iodine 131 in Differentiated Thyroid Cancer Patients

**DOI:** 10.3390/genes17060645

**Published:** 2026-05-31

**Authors:** Roko Granić, Ivan Blažeković, Josipa Miš, Ivan Šamija, Tihana Regović Džombeta, Gorana Mirošević, Denis Bakunić, Kristina Kralik, Ana Fröbe, Zvonko Kusić, Tomislav Jukić

**Affiliations:** 1 Department of Oncology and Nuclear Medicine, University Hospital Center Sestre Milosrdnice, 10 000 Zagreb, Croatia; blazekovic90@gmail.com (I.B.); ana.frobe@kbcsm.hr (A.F.); 2 School of Medicine, Catholic University of Zagreb, 10 000 Zagreb, Croatia; 3 Department of Clinical Chemistry, University Hospital Center Sestre Milosrdnice, 10 000 Zagreb, Croatia; jperisa92@gmail.com (J.M.); ivan.samija@kbcsm.hr (I.Š.); 4 School of Dental Medicine, University of Zagreb, 10 000 Zagreb, Croatia; gorana.mirosevic@kbcsm.hr; 5 Department of Pathology Ljudevit Jurak, University Hospital Center Sestre Milosrdnice, 10 000 Zagreb, Croatia; tihana.dzombeta@mef.hr; 6 School of Medicine, University of Zagreb, 10 000 Zagreb, Croatia; denis.bakunic16@gmail.com; 7 Department of Internal Medicine, University Hospital Center Sestre Milosrdnice, 10 000 Zagreb, Croatia; 8 Faculty of Medicine Osijek, Josip Juraj Strossmayer University of Osijek, 31 000 Osijek, Croatia; kristina.kralik@gmail.com; 9 Croatian Academy of Sciences and Arts, 10 000 Zagreb, Croatia; zaklada@hazu.hr

**Keywords:** differentiated thyroid cancer, iodine-131, *BRAF^V600E^*, *TERT* promoter, mutation, treatment response, prognosis

## Abstract

**Objectives:** The response to radioiodine therapy (RAI) in differentiated thyroid cancer (DTC) patients is one of the most important factors that determines the treatment outcome and overall prognosis. The objective of this study is to determine the correlation between *BRAF^V600E^* and *TERT* promoter (*TERTp*) mutation in tumor samples of DTC patients with the response to RAI as well as correlation with clinical and pathohistological features. **Methods:** Samples of 110 DTC patients (80 with intermediate and high risk of disease recurrence-IHR and 30 with distant metastases) were analyzed for *BRAF^V600E^* and *TERTp* mutation (*BRAF/TERT*) and 89 patients were assessed for the response to RAI. **Results:** Sixty-one (55.5%) patients had *BRAF^V600E^* mutation, 30 (27.3%) had *TERTp* mutation, while 21 (19.1%) patients had both mutations. In the IHR group, the study showed a statistically significant association between genotype *BRAF/TERTp* and treatment outcome (*p* = 0.04). IHR DTC patients with *BRAF-/TERT-* finding showed in general an excellent response to RAI, while patients with *BRAF/TERT* co-mutation had a predominantly incomplete or indeterminate response. In DTC patients with *BRAF/TERT* co-mutation that presented with distant metastases, a tendency to a higher frequency of RAI-refractory (RAI-R) disease was recorded, but without statistical significance. The pathohistological and clinical features that significantly correlated with *BRAF/TERT* status are age at diagnosis, locoregional lymph node involvement, the largest positive lymph node diameter, tumor angioinvasion and the presence of distant metastases. **Conclusions:** *BRAF/TERT* co-mutation may be associated with a less favorable disease course and poorer response to RAI. However, findings in patients with distant metastases should be considered exploratory due to the limited sample size.

## 1. Introduction

Thyroid cancer (TC) is the most common endocrine tumor that affects predominantly women (3:1) and, due to its high incidence worldwide, represents a major challenge to modern medicine, causing increasing pressure on the health care systems of many countries. More than 90% of TCs present as DTC, which includes papillary, follicular and oncocytic subtypes and is associated with good prognosis. Less frequent TCs are poorly differentiated TC as well as medullary and anaplastic TC, which have an unfavorable or even fatal prognosis.

Globally, in the last 5 years, the age-standardized incidence rates of thyroid cancer were around 10 per 100,000 women and 3 per 100,000 men, but age-standardized mortality rates have stayed continuously low, around 0.5 per 100,000 women and 0.3 per 100,000 men. When incidence rates in countries are stratified using the Human Development Index, it is observed that the incidence rates in both sexes are five times higher in highly developed countries than in low- and medium-developed countries, probably due to improved diagnostics, reflecting health care accessibility. However, mortality rates are relatively similar across different settings [1].

According to Globocan Cancer Observatory data, Croatia is rated as the fourth country in Europe and the eleventh country in the world regarding the incidence of TC [2]. According to the data of the Croatian Public Health Institute Cancer Registry for the year 2023, TC is the fifth most common cancer in the female population (around 5 percent of all new cancer cases reported) and the most common endocrine cancer in both sexes. The incidence rate of TC in Croatia is 33.1 and 11.6 per 100 000 in women and men, respectively. The latest data reported a slight increase in the number of new cases per annum (876 in 2023, as opposed to 843 in 2019) [3].

This current TC epidemiological landscape is strongly suggestive of a large effect of overdiagnosis in many countries and settings worldwide. Recently, a slight increase in mortality rates can be attributed to the changes in the nature of the disease, i.e., more aggressive subtypes that may be linked to certain environmental factors that affect oncogenesis [4].

Apart from the fact that most TCs have an indolent nature and present with locoregional disease that invades only structures in the neck, predominantly local lymph nodes, a certain percentage of TCs, even those well-differentiated types, can present with hematogenous metastases—dissemination to distant organs and lymph nodes. Around 4% of DTC patients present with distant metastases. Herewith, the recurrence and the disease mortality rates can increase significantly, affecting the quality of life of DTC patients.

Distant metastases (DM) in DTC patients can be found in approximately 3.5–15% of cases, the most common site being the lung (49%), followed by the bone (25%), whereas simultaneous pulmonary and bone metastases occur in around 15% of cases affecting the spine (34.6%), pelvis (25.5%), sternum and ribs (18.3%) and extremity bones (15.6%), often in areas with rich blood flow. Distant metastases are more prevalent in follicular thyroid cancer (FTC) (7–28%) than in papillary thyroid cancer (PTC), where they range from 1.4 to 7%. Other rare or relatively rare sites of DM include the brain, eye, breast, liver, kidney, muscle and skin [5,6,7,8,9].

It is important to note that age is an important prognostic factor in DTC patients—especially in DTC patients older than 60 years, whose survival rate is usually significantly reduced due to diagnostic issues, advanced disease at diagnosis, and associated comorbidities—and not to underestimate the significance of a probably different genetic setup of the disease in that population [5].

Therefore, it would be very advantageous to determine certain molecular markers that could stratify patients with a higher probability of the recurrence or distant spread of the disease. As DTCs as a rule react very well to RAI, i.e., accumulate radioiodine that eventually destroys tumorous cells, it would be useful if those molecular markers could also predict response to RAI, e.g., stratify patients who will benefit from RAI versus patients at risk of becoming radioiodine refractory.

Radioiodine-refractory DTC (RAI-R-DTC) is difficult or even impossible to treat with radioiodine because of the absence of the sodium iodide transporter in the basal membrane of thyroid follicular cells that is necessary for iodine uptake. The usual causes of this occurrence are the mutations or rearrangement of genes causing the aberrant activation of signaling pathways, resulting in abnormal expression of thyroid-specific genes, leading to resistance of DTC cells to RAI [10].

The 2015 and 2025 American Thyroid Association (ATA) guidelines [11,12] classify RAI-R-DTC into four categories. The first is primary RAI-R-DTC, where tumor cells are unable to concentrate radioactive iodine at all. In the second type, the cells lose the ability to concentrate radioactive iodine later in the course of the disease; the third type shows tumor heterogeneity, i.e., some metastatic sites show good uptake of radioiodine while in other sites, the uptake is poor or nonexistent. In the last category of RAI-R-DTC, the disease progresses despite the apparently good uptake of radioiodine in known metastatic sites [13]. The 10-year survival rate for RAI-R-DTC is reduced to 10–63%, with a median overall survival after RAI-R diagnosis of 8.2 years [10,14].

### BRAF and TERT Gene Mutation in Thyroid Cancer

The most common *BRAF* mutation is *V600E*, which constitutes almost 90% of all *BRAF* mutations in TC [15]. It was first identified in 2003 [16], with current prevalence in TC of approximately 45–50%. The prevalence ranges from 27 to 87% in PTC and from 25 to 30% in anaplastic thyroid carcinoma (ATC), and it is very uncommon or almost absent in FTC as well as in benign thyroid tumors [15]. The *BRAF* gene encodes B-raf protein, a serine/threonine protein kinase, mitogen-activated protein kinase kinase kinase (MAPKKK) in the mitogen-activated protein kinase (MAPK) signaling pathway [10]. The most common mutation in the *BRAF* gene is the missense nucleotide substitution c. 1799 T>A, a transverse point mutation of the 15th exon. It results in the conversion of B-raf protein residue 600 from valine to glutamic acid (V600E) [17]. The *BRAF^V600E^* mutation causes loss of the inhibitory loop of the constitutive serine/threonine kinase activity and allows B-raf^V600E^ to activate itself and the MAPK signaling pathway [18]. *BRAF^V600E^*-mutated DTC cells exhibit uncontrolled cell proliferation, migration, and invasion as well as reduced expression of genes involved in iodine uptake and metabolism. The association between the presence of the *BRAF^V600E^* mutation and clinicopathological prognostic factors of PTC has been widely investigated over the last 20 years.

Although the association with adverse pathological features such as lymph node metastases and extrathyroidal extension, as well as increased recurrence risk in PTC, was consistently shown in the studies and meta-analyses, the clinical utility of *BRAF^V600E^* mutation as a sole prognostic marker remains limited and is not widely accepted [15,19]. The possible correlation between the *BRAF^V600E^* mutation and DTC RAI refractoriness was first described in 2005. Recent studies have revealed the molecular mechanisms that determine the progression and aggressiveness of PTC due to *BRAF* mutation, which include the down-regulation of major tumor suppressor genes and thyroid iodide-metabolizing genes. It is also observed that *BRAF* mutation in TC causes up-regulation of cancer-promoting molecules, such as vascular endothelial growth factor (VEGF) A and its receptor VEGF receptor (VEGFR)-2, the main regulators of the angiogenic process [20]. This happens through suppression of the expression of genes that are involved in iodine metabolism, impairing the efficiency of RAI. Despite reduced RAI avidity, some studies suggest that RAI treatment in *BRAF^V600E^*-positive PTC should be regularly performed, as complete loss of RAI uptake is very uncommon [21]. Therefore, it has to be emphasized that rather than being an all-or-nothing phenomenon, refractoriness to RAI appears to be a spectrum, so the loss of iodine uptake in the majority of thyroid tumors is only partial. *BRAF^V600E^*-mutated PTCs are highly heterogeneous with diverse epigenetic, genetic and differentiation profiles [15].

The therapeutic role of *BRAF* uses the possibility of specific inhibitors to disrupt MAPK signaling pathways in *BRAF*-mutated cancer cells as a new strategy for the treatment of thyroid cancer. Some of those pharmacological inhibitors are selumetinib, vemurafenib and dabrafenib, which have been clinically tested in *BRAF*-mutant TC patients. The aforementioned inhibitors act as antitumor agents as well as redifferentiation agents to enhance the efficiency of RAI by resensitizing *BRAF*-mutant tumors to RAI. In that way, tumor regression and disease control can be obtained in previously RAI-R tumors that can again be treated with RAI [21].

Another potential genetic biomarker that is being investigated is the mutation of the promoter region of the *TERT* gene. In the presence of a mutation in the promoter region of the *TERT* gene, telomerase suppression is inhibited in reactivated somatic cells (proliferating and dedifferentiated tumor cells). *TERT* is the catalytic subunit of telomerase, an enzyme responsible for maintaining chromosomal integrity and genome stability. In normal cells, telomeres shorten in time with each cell division, eventually leading to cellular aging. Telomerase reactivation prevents its shortening, thus contributing to cancer cell immortality. *TERT* plays a significant role in telomerase activation in malignant cell transformation and in the regulation of genes that are involved in cellular growth [22]. Some studies suggest that *TERT* can function as an oncogene, with promoter mutations of the *TERT* gene potentially playing a substantial role in thyroid cancer [23]. *TERT* mutation does not directly reduce the accumulation of RAI in tumor cells, but it affects the proliferative immortality of the cell—a cell that focuses on proliferation usually neglects the function (e.g., the accumulation of iodine in the cell via the Na/I symporter).

In a 2020 meta-analysis conducted by a group of Chinese authors that collected data from 51 studies with 11,382 DTC cases, the frequencies of *TERT* promoter mutation ranged from 2.1 to 75%, and the overall average frequency was 10.9%. The average frequency of *TERT* promoter mutation in PTC was 10.6%, compared to 15.1% in FTC [24].

In another study (Nhung et al., 2025), the results of 2092 patients were analyzed and showed that 3.4% of DTC patients had *TERT* promoter mutations [25]. The frequency of *TERT* promoter mutations was only 0.5% in papillary thyroid microcarcinoma (PTMC) ≤ 1 cm and 5.8% in papillary thyroid carcinoma (PTC) > 1 cm. The frequency of *TERT* promoter (*TERTp*) mutations was significantly associated with older age, the size of the primary tumor and aggressive histological type (follicular, poorly differentiated and anaplastic thyroid carcinoma). In *TERT*-mutated thyroid cancers, advanced T stage, advanced N stage and distant metastasis at diagnosis were more common [25].

The frequency of *BRAF* and *TERT* co-mutation in DTC varies but generally shows a co-occurrence rate of around 3–10% of all DTC cases, but is observed to be higher in RAI-R types [26].

The frequency of *TERTp* mutation in the primary tumor, local lymph node metastases and distant metastases increases from 10.5% in the primary tumor and 12.9% in local lymph nodes to 52.4% in distant metastases. However, *BRAF^V600E^* mutation frequency decreases from 44.6% in the primary tumor and 41.7% in lymph node metastases to 23.8% in distant metastases, as presented by Melo et al. in their study on 437 tissue samples [27]. Therefore, *TERTp* mutation may play a role in the occurrence of distant metastases [25].

DTC patients with *BRAF^V600E^* mutation are more likely to also have the *TERTp* mutation. *TERTp* mutation was an independent predictive factor for poor prognosis in PTC patients, but the predictive value of *BRAF^V600E^* mutation remains inconclusive. Patients with both mutations have remarkably higher risks of adverse outcomes compared with those with a single mutation [25,26,27,28,29], as presented by Chen et al. with the statement: *BRAF+TERT+* > *BRAF-TERT+* > *BRAF+TERT-* [29].

Moreover, the combination of *BRAF^V600E^* and *TERTp* mutation has a strong negative synergistic effect considering the recurrence of the disease, mortality rates and overall poorer prognosis in DTC. Recent studies have confirmed that the *BRAF^V600E^* and *TERTp* co-mutation is also strongly associated with radioiodine resistance and impaired iodine receptor metabolism in recurrent DTC, thus serving as a biomarker for potential or even probable radioiodine treatment failure in those tumors [25,26,30,31].

Today’s official guidelines of national and international thyroid societies do not yet fully include the above-mentioned mutations as a strong criterion for categorization of the disease, especially in relation to iodine-131 treatment response, mostly due to the lack of clear evidence, although they recognize their potential value. Despite previous research on the existence and influence of *BRAF* and *TERT* mutations on DTC and the response to RAI, the area of genetic mutations in the process of the onset of the disease is still under investigation and awaits further confirmation [32,33,34].

The aim of this study is to determine the correlation between *BRAF^V600E^* and *TERT* promoter (*TERTp*) mutation analysis in tumor samples of DTC patients with high and intermediate risk of disease recurrence according to ATA guidelines [11,12], as well as in DTC patients with distant metastases, with the response to RAI as well as with clinical and pathohistological features.

This could enable early selection of patients with a need for more aggressive treatment (e.g., higher iodine-131 therapeutic activity) and a more frequent follow-up approach versus patients who will have a good response to RAI and thus a more favorable outcome.

Treatment with RAI includes ablation/destruction of the remnant thyroid tissue in the thyroid bed, adjuvant therapy to destroy potential microscopic tumor cells and lower the rate of tumor recurrence, and treatment of known residual or recurrent disease: locally invasive tumor, neck lymph node metastases or distant metastases.

In the group of patients with intermediate and high risk for disease recurrence (according to ATA guidelines), there is a need for molecular prognostic markers to assess response to RAI as the main therapy in DTC patients, together with surgery. Iodine I-131 refractoriness usually indicates tumor dedifferentiation and a less favorable outcome in these patients with development of regional and/or distant metastases.

We hypothesize that DTC patients in the group of intermediate and high risk of disease recurrence who are carriers of *BRAF^V600E^* and *TERTp* mutations might have a worse response to RAI and thus a less favorable outcome.

## 2. Materials and Methods

### 2.1. Participants

In this retrospective study, we collected data from 110 adult patients diagnosed with DTC, treated at the Department of Oncology and Nuclear Medicine, Sestre Milosrdnice University Hospital Center Zagreb, the Referral Center for Thyroid Diseases of the Ministry of Health of the Republic of Croatia. A formal sample size calculation was not performed due to the retrospective design and fixed number of available cases. Response to RAI was assessed in 89 patients (80.9%; 95% CI 72.6–87.2%) due to a complete set of data. There were no significant differences between the included and excluded cohorts apart from a lack of follow-up data that prevented us from determining those patients’ response to RAI. All procedures that we conducted were in accordance with the 1964 Helsinki Declaration and its later amendments, or similar applicable ethical standards. The study has been approved by our institution’s Ethics Committee (approval No. EP-8247/19-5). Informed consent was signed by all participants in the study. We collected paraffin blocks with incased primary thyroid tumor that were stored in the Department of Pathology after total thyroidectomy. The data regarding the pathohistological (PH) characteristics of the primary tumor, patients’ general data, as well as data related to postoperative oncological treatment and follow-up were assessed.

From the PH findings we gathered the following information: primary tumor size, localization of the tumor within the thyroid (left or right lobe, isthmus), tumor dissemination within the thyroid gland, infiltration of the thyroid capsule, extrathyroidal extension, and angioinvasion (i.e., tumor cells in vascular spaces determined by histopathologic examination). We also assessed the TNM classification of the disease at the time of thyroidectomy, the type of DTC (papillary or follicular) and the presence of neck and distant metastases.

The study group that we tested for *BRAF^V600E^* and *TERTp* mutations consisted of 62 female (56%) and 48 male (44%) participants. The overall mean age of the participants at the time of diagnosis was 55 y (24–75 y), with the oldest patients coming from the group of patients with *BRAF/TERT* co-mutation (mean age 66 y), and the youngest from the group with neither of the mutations (mean age 31 y).

The patients enrolled in this study were intermediate- and high-risk DTC patients who are usually treated with RAI. They underwent genetic testing for the presence of *BRAF^V600E^* and *TERTp* (C228T and C250T) mutations. According to ATA Estimated Risk of Structural Recurrence patient stratification, low-risk DTC patients (with a recurrence rate of less than 10%) are those with a tumor diameter ≤ 4 cm (T1 and T2), unifocal tumor, ≤5 lymph nodes < 2 mm affected (pN0a or cN0 and pN1a), and negative or microscopic infiltration of surgery margins. Low-risk FTC patients are only those that are minimally invasive, i.e., that have only capsular invasion. The intermediate-risk DTC patient group (recurrence rate from 10 to 30%) comprises T1, T2 and T3 tumors with any of the following: unilateral or bilateral multifocality, microscopic extrathyroidal extension, aggressive histology, vascular invasion or clinically evident bilateral lymph node metastases, or more than 5 lymph nodes affected (cN1a, cN1a > 2 mm or cN1b < 3 cm). Intermediate-risk FTC patients are those that, apart from the aforementioned characteristics, have a limited vascular invasion of less than 4 vessels. High-risk DTC patients, with a recurrence rate of more than 30%, are those with T3a tumors with microscopic extrathyroidal extension, T3b or T4 tumors, or any T tumors with any of the following characteristics: poorly differentiated or high-grade tumors, gross incomplete resection, lymph nodes affected ≥3 cm (cN1 ≥ 3 cm), extranodal extension or distant metastases. High-risk FTCs are encapsulated angioinvasive tumors or FTCs with extensive vascular invasion of more than 4 vessels [11,12].

The parameters used to assess the response to RAI included prior and posttreatment determination of stimulated serum levels of tumor marker thyroglobulin (Tg), as well as levels of antithyroglobulin antibodies (TgA), and imaging findings: whole-body iodine-131 scans (I-131 WBS) and neck ultrasound (US) with or without fine needle aspiration biopsy (FNAB). To assess the response to RAI, ESMO criteria (European Society for Medical Oncology) were used, with 4 categories of response to radioiodine therapy: (1.) excellent response (negative WBS/US findings, undetectable TgA and stimulated Tg <1 ng/mL); (2.) incomplete biochemical response (negative WBS/US findings, stimulated Tg >= 10 ng/mL or increased TgA); (3.) incomplete structural response (residual disease on WBS or neck ultrasound, regardless of Tg or TgA); and (4.) indeterminate response (nonspecific WBS/US findings or weak accumulation of iodine 131 activity in the thyroid bed/stimulated Tg 1–10 ng/mL or TgA stable or decreasing in patients without imaging evidence of disease) [32,33].

Patients with distant metastases (23 subjects out of 89 assessed) have been analyzed separately, because these patients usually have a different course of the disease and their response to RAI has to be evaluated according to modified criteria. The response to RAI in patients with distant metastases (high-risk group) has been assessed after at least two RAI applications or after they have received a cumulative dose of 600 mCi of iodine-131. These patients were classified either as refractory to iodine-131 (RAI-R-DTC) or as patients with controlled disease (reduction in stimulated Tg by at least 20% after the first two applications of RAI associated with distant metastases accumulating iodine-131) [12,33].

### 2.2. Histological Analysis of the Primary Tumor

The existence of mutations in the promoter region of the *TERT* gene and the V600E mutation in the *BRAF* gene has been analyzed in the tissue samples of the primary DTC tumor.

DNA was isolated from the collected PH samples of the primary tumor, which, due to the quality of the samples, should not have been older than ten years, i.e., from samples collected between 2011 and 2021, using commercial DNA isolation kits. The quality of the isolated DNA was validated by standard methods.

In order to detect *TERTp* mutations, the *TERTp* region was amplified by PCR amplification and, after checking the appropriate sizes of the prepared amplicons, analysis was done by Sanger sequencing. Determination of the *BRAF^V600E^* mutation was performed by real-time polymerase chain reaction using specific TaqMan probes. The criteria of good laboratory practice were followed during sampling, isolation and analysis.

The removed thyroid tissue containing the tumor was embedded in paraffin blocks after fixation in 10% buffered formalin and dehydration in increasing concentrations of alcohol. Paraffin blocks were cut in 5 µm sections. Deparaffinization in xylene and hemalaun and eosin staining (H&E) were performed with the standard method. The sections that were cut from each paraffin block were then used for the extraction of genomic DNA for *BRAF^V600E^* and *TERTp* mutation analysis.

### 2.3. DNA Extraction and BRAF^V600E^/TERTp Mutation Detection

DNA was extracted by following the manufacturer’s instructions using the QIAamp DNA FFPE Tissue kit (Qiagen, Hilden, Germany). The concentration and quality of the extracted DNA were determined using a NanoDrop 2000 spectrophotometer (Thermo Fisher Scientific, Waltham, MA, USA). The only DNA that was used for further processes was one that had an OD (optical density coefficient, 260/280) of 1.70 ± 20%.

### 2.4. BRAF^V600E^ Mutation Detection

For *BRAF* mutation analysis, target DNA was amplified and detected using a commercially available *BRAF^V600E^* TaqMan^®^ Mutation Detection Assay with predesigned allele-specific probes and primers (Applied Biosystems, Foster City, CA, USA) on a 7500 Real-Time PCR System (Applied Biosystems, Foster City, CA, USA), following the manufacturer’s instructions. Prior to analysis, extracted DNA was pre-diluted to a concentration of 6.25 ng/µL. Each sample was analyzed in two reactions using TaqMan^®^ Mutation Detection Assays (Applied Biosystems, Foster City, CA, USA): one with allele-specific probes for detection of the *BRAF^V600E^* mutation (*BRAF*_476_mu) and one for the reference genotype (*BRAF* rf). The reaction mixture contained 10 µL TaqMan^®^ Genotyping Master Mix, 4 µL DNA, 4 µL PCR-grade water, and 2 µL allele-specific probe solution. Real-time PCR cycling conditions consisted of 95 °C for 10 min (enzyme activation), followed by 5 cycles of 92 °C for 15 s (DNA denaturation) and 58 °C for 1 min (annealing/elongation). The subsequent 40 cycles differed slightly, with 95 °C for 15 s for DNA denaturation and 60 °C for 1 min for annealing and elongation. Positive and negative controls were included in each run. Competitive Allele-Specific TaqMan PCR enables detection of 0.1% mutant alleles.

### 2.5. TERT Promoter Mutation Detection

Detection of *TERT* promoter mutations was more challenging due to the high GC content of this region (over 80%). To address this issue and to increase the specificity of the sequenced region, nested PCR was performed using the AmpliTaq Gold™ 360 DNA Polymerase Amplification Reagent Kit (Applied Biosystems, Foster City, CA, USA), which includes a GC enhancer additive. The nested PCR was designed as follows: the forward primer (F1) used in the first reaction was 5′-AGTGGATTCGCGGGCACAGA-3′; the forward primer (F2) used in the second reaction was 5′-GTCCTGCCCCTTCACCTTC-3′; and a shared reverse primer (R) was used in both reactions: 5′-CAGCGCTGCCTGAAACTC-3′. For a single sample, the first-round PCR master mix contained 2.5 µL buffer (1×), 1.5 µL MgCl_2_ (1.5 mM), 0.5 µL dNTP mix (0.2 mM), 2 µL primer F1 (1 µM), 2 µL primer R (1 µM), 1.25 µL GC enhancer (5%), 0.2 µL enzyme (1 U), and 10.05 µL PCR-grade water. DNA was diluted to 20 ng/µL using the elution buffer from the DNA extraction kit, and 5 µL (100 ng) was used as a template in the first PCR reaction.

The cycling conditions were as follows: initial hot-start activation at 95 °C for 10 min, followed by 40 amplification cycles of 30 s at 95 °C (denaturation), 30 s at 57 °C (annealing), and 30 s at 72 °C (extension), ending in final extension at 72 °C for 7 min. For the second-round (nested) PCR, the master mix contained double the amounts of the reagents used in the first PCR, replacing primer F1 with F2 and increasing the enzyme to 0.25 µL (1.25 U). To reach a final reaction volume of 50 µL, 2 µL of first-round PCR product and 27.5 µL PCR-grade water were added. Cycling conditions were identical to the first PCR, except that the number of amplification cycles was reduced to 30. Final product size was estimated to be 162 bp long. PCR products were size-verified by electrophoresis on a 2% agarose gel (Sigma-Aldrich, St. Louis, MO, USA) containing SYBR Safe DNA Gel Stain (Thermo Fisher Scientific, Waltham, MA, USA) and compared to a DNA molecular weight marker VIII (Roche, Basel, Switzerland). Gels were run at 90 V for 40 min and visualized under UV light. The expected product size was 162 bp. The DNA concentration of the second PCR was measured using the Qubit dsDNA BR Assay Kit (Thermo Fisher Scientific, Waltham, MA, USA), diluted to 1.5 ng/µL with PCR-grade water, and submitted to Macrogen (Basel, Switzerland) for Sanger sequencing using the nested primers with the “Difficult Sequencing” option enabled. For the detection of *TERT* promoter mutations, we used Sanger sequencing, which can reliably detect 15–20% mutant alleles. This sensitivity is adequate for the detection of clonal somatic heterozygous mutations in DNA isolated from cancer tissue but may miss subclonal mutations, which is a limitation of our study. The method we used, Sanger sequencing, was used in the majority of published studies analyzing *TERT* promoter mutations in thyroid cancer.

*TERT* promoter mutation results obtained from Sanger sequencing were analyzed using software Clustal*X* (version 2.1) and Benchling (Benchling, San Francisco, CA, USA) and categorized according to the presence of the C228T and C250T mutations.

### 2.6. Statistical Methods Employed in Data Processing

Categorical variables were presented as absolute and relative frequencies. Differences between categorical variables, i.e., the association of the response to RAI as well as some clinicopathological features between the groups with and without *BRAF^V600E^* and *TERTp* mutation and *BRAF^V600E^/TERT* co-mutation, were assessed using the chi-square test, with Cramer’s V calculated as a measure of effect size. The normality of continuous variables was evaluated using the Shapiro–Wilk test. Continuous variables were described using the median and interquartile range (IQR). Differences in continuous variables across *BRAF/TERT* mutation groups were analyzed using the Kruskal–Wallis test, followed by Conover’s post hoc test. All *p* values were two-sided, and the level of significance (statistically significant result) was set at alpha (α) = 0.05. Statistical analyses were performed using MedCalc^®^ Statistical Software, version 23.4.8 (MedCalc Software Ltd., Ostend, Belgium; https://www.medcalc.org). The study report was prepared in accordance with the reporting guidelines for biomedical and health research provided by the EQUATOR Network.

## 3. Results

Out of the 110 participants, we found that 61 had *BRAF^V600E^* mutation (55.5%; 95% CI 46.1–64.4) and 30 patients (27.3%; 95% CI 19.8–36.3) had *TERTp* mutation. The co-mutation (*BRAF^V600E^ + TERTp*) was present in 21 patients (19.1%; 95% CI 12.8–27.4). The distribution of mutation combinations showed that 40 patients (36.4%; 95% CI 28.0–45.7) had *BRAF+/TERT-*, 9 (8.2%; 95% CI 4.4–14.8) had *BRAF-/TERT+*, and 40 (36.4%; 95% CI 28.0–45.7) had neither mutation (*BRAF-/TERT-).* When the intermediate- and high-risk groups were analyzed separately, 45.0% (36/80) of patients had only *BRAF^V600E^* mutation (*BRAF+/TERT-*), as opposed to 1.3% (1/80) with only *TERTp* mutation (*BRAF-/TERT+*). In the group of patients with distant metastases, the most frequent mutation profile was *BRAF-/TERT-* (11/30; 36.7%), followed by the *BRAF-/TERT+* mutation profile (8/30; 26.7%) and the *BRAF/TERT* co-mutation profile (7/30; 23.3%) (Table 1). In 30 patients who had distant metastases at the time of diagnosis, more tested positive for *TERTp* mutation (15/30; 50%) than for *BRAF^V600E^* mutation (11/30; 36.7%). However, in the intermediate- and high-risk patient group (80 patients), we detected the reverse situation—the predominant mutation was *BRAF^V600E^* (50/80; 62.5%), against 18.8% (15/80 patients) who had *TERTp* mutation. Forty out of the 110 patients tested had neither of the aforementioned mutations (36.4%); that percentage is almost equally divided between the intermediate- and high-risk group and the distant metastases group (36.2% and 36.7%, respectively). It is expected that the mutational burden is higher in the intermediate- and high-risk group of patients as opposed to the low-risk patient group.

The distribution of *BRAF/TERT* mutations differed significantly according to metastatic status. In patients with local disease, the most common was *BRAF+/TERT-*, present in 36/40 (90%) patients, while in patients with distant metastases, *BRAF-/TERT+* was most common (8/9 subjects, 89%) (χ^2^ test, *p* < 0.001; Cramer’s V = 0.464) (Table 2).

Significant differences in the distribution of BRAF/TERT mutations were also noted according to lymph node involvement (N) classification, where patients without involved lymph nodes (N0) most often had *BRAF-/TERT-* status, while in the group with positive lymph nodes (N1 and N1a), the *BRAF+/TERT-* status was significantly more frequent (χ^2^ test, *p* = 0.01; Cramer’s V = 0.264) (Table 2).

According to the M classification (presence of distant metastases), patients without confirmed distant metastases at the time of surgery most often had *BRAF+/TERT-* status (92%), while in patients with distant metastases, *BRAF-/TERT+* status was present in all seven (100%) cases (χ^2^ test, *p* < 0.001; Cramer’s V = 0.522) (Table 2).

Analysis of histopathological features showed that angioinvasion was significantly more common in patients with *BRAF-/TERT*+ status, recorded in four (44%) cases (χ^2^ test, *p* = 0.02; Cramer’s V = 0.304) (Table 2).

Patients with pathohistologically confirmed neck lymph node metastases had *BRAF+/TERT-* status significantly more often (97%) (χ^2^ test, *p* < 0.001; Cramer’s V = 0.411), while *BRAF-/TERT+* status was dominant in patients with distant metastases, present in eight (89%) cases (χ^2^ test, *p* < 0.001; Cramer’s V = 0.499) (Table 2).

A statistically borderline difference (*p* = 0.05) was found between the groups of patients with unilateral disease (involvement of only one lobe or isthmus of the thyroid) and bilateral disease, where the tumor was detected in multiple regions of the thyroid (both lobes or lobe and isthmus of the thyroid) according to the presence of *BRAF/TERT* mutations.

Gender, pathohistological subtype (papillary/follicular), lobe involvement, extrathyroidal extension, tumor size, lymph node metastases (LNM) with extranodal extension, and postoperatively proven neck metastases did not show statistically significant differences in the distribution of *BRAF/TERT* mutations (Table 2).

The age of patients differed significantly between mutation groups. Patients with *BRAF/TERT* co-mutation were significantly older, while patients with *BRAF-/TERT-* status were significantly younger (Kruskal–Wallis test, *p* < 0.001).

Significant differences were also noted in the diameter of the largest positive lymph node between mutation groups (Kruskal–Wallis test, *p* = 0.005), with a smaller diameter in the *BRAF+/TERT-* mutation group.

The diameter of the largest tumor lesion and the cumulative dose of radioactive iodine did not differ significantly between the mutation groups, neither in the intermediate- and high-risk groups nor in patients with distant metastases (Table 3).

### Radioiodine Therapy and BRAF/TERT Status

In DTC patients with intermediate and high risk of disease recurrence, the distribution of response to RAI, according to the presence of *BRAF^V600E^* and *TERTp* mutations, showed a statistically significant association between genotype and treatment outcome categories (Fisher’s exact test, *p* = 0.04). The *BRAF-/TERT-* group more frequently achieved a complete (excellent) response, while the group with *BRAF/TERT* co-mutation had less favorable therapeutic outcomes (Table 4).

However, in patients with distant metastases, no statistically significant association was found between *BRAF* or *TERT* mutation status and the incidence of RAI-R disease (Fisher’s exact test). Although the group with *BRAF/TERT* co-mutation had a higher proportion of RAI-R disease, the differences did not reach statistical significance. Mutation-negative subjects predominantly had controlled disease (Table 5).

Multivariate logistic regression analysis including age, *BRAF/TERT* co-mutation status, and the presence of distant metastases showed that distant metastases were independently associated with non-excellent response to RAI (OR 0.21). *BRAF/TERT* co-mutation status showed a non-significant trend toward association with non-excellent response (Table 6).

## 4. Discussion

In the intermediate- and high-risk patient group, 45% had a solitary *BRAF^V600E^* mutation, as opposed to only 1.3% of *TERTp*-positive patients, which is in accordance with most authors [16,17,25]. In the group of intermediate- and high-risk patients, the most common mutation profile was *BRAF+/TERT-*, present in 90% of patients, as opposed to the group of patients with distant metastases, with the most common mutation profile, *BRAF-/TERT+*, recorded in 89% of patients. Therefore, in our study, the *BRAF-/TERT+* mutation profile seems to be associated with distant spread of DTC linked to poor outcome. Previous studies linked *TERTp* mutation with distant metastases of DTC [27,30]. The correlation between the mutational status and risk stratification group could therefore be observed and is statistically significant according to our data (*p* < 0.001).

In DTC patients with intermediate and high risk of disease recurrence, our data showed a statistically significant association between genotype (*BRAF^V600E^/TERTp* status) and treatment outcome categories (Fisher’s exact test, *p* = 0.04). However, given the small sample size and low cell counts in several subgroups, this finding should be interpreted with caution, as the statistical significance may be unstable and sensitive to small changes in the data. To further assess the independence of this association, a multivariate logistic regression analysis was performed. In this analysis, only the presence of distant metastases remained an independent predictor of non-excellent response to RAI, while *BRAF/TERT* co-mutation status showed a non-significant trend toward association. This suggests that part of the observed bivariate association between *BRAF/TERT* status and treatment response may be influenced by disease extent, particularly the presence of distant metastases. However, due to the limited sample size, the multivariate model was restricted to a small number of variables. Patients with *BRAF-/TERT-* status generally showed an excellent response to RAI, while those with *BRAF/TERT* co-mutation more frequently had an incomplete or indeterminate response. The relatively wide confidence intervals and borderline statistical significance observed in some analyses reflect the limited sample size and should therefore be interpreted with caution. These findings are consistent with previous studies reporting a negative synergistic effect of *BRAF/TERT* co-mutation on disease aggressiveness, recurrence, and overall prognosis in DTC [25,30,31]. The presence of a single mutation is therefore more favorable than the presence of co-mutation, with *TERTp* mutation alone being linked with worse outcome than *BRAF^V600E^*. Chen et al. showed stratification with the following scheme: *BRAF+TERT+* > *BRAF- TERT+* > *BRAF+ TERT-* [30]. In our study, intermediate- and high-risk patients had an incomplete response to RAI, either biochemical or structural, in almost 50% of cases, while the other 50% had an excellent response. The criteria for incomplete and indeterminate response are rather strict (according to ESMO criteria and ATA guidelines, i.e., undetectable Tg levels and no remnant thyroid tissue in the thyroid bed), so almost all patients can be considered as having an excellent or at least acceptable long-term response to RAI. Almost a quarter of patients with distant metastases had RAI-R disease, but most, i.e., three-quarters of patients, had controlled disease. In our study, in the group of patients with distant metastases, the correlation between *BRAF/TERT* mutational status and response to RAI was not statistically significant. As previously stated, refractoriness to RAI is not a straightforward all-or-nothing phenomenon, but rather a spectrum of tumor iodine uptake. Therefore, the loss of iodine uptake in the majority of DTCs is only partial [21]. However, the group with *BRAF/TERT* co-mutation had a higher proportion of RAI-R disease, similar to Nhung et al.’s study. The coexistence of *BRAF* and *TERT* mutation can be associated with the higher risk of radioiodine avidity loss [25].

Pathohistological and clinical characteristics of our patients were analyzed mostly by their TNM (Tumor-lymph Node-distant Metastasis) status. It can be observed that, considering the size of the largest tumor lesion (T status), the predominant status was T3, as is expected in the intermediate- and high-risk patients. Although a higher proportion of T3 tumors was observed in the *BRAF/TERT* co-mutation group and in the *BRAF-/TERT+* group, this difference did not reach significance (*p* = 0.09) and should be interpreted as an observational trend. Similarly, the *BRAF-/TERT+* group showed a tendency toward larger average tumor size, while the *BRAF+/TERT-* group had smaller tumors; however, these differences were not statistically significant (*p* = 0.43) and should be interpreted with caution.

Lymph node involvement (N status), however, showed that *BRAF+*/*TERT-* mutational status was significantly more common in the N1 and N1a group of patients (patients with lymph node involvement), and patients with neither of the mutations (*BRAF-/TERT-*) predominantly had no neck lymph node involvement (*p* = 0.01). The diameter of the largest positive lymph node showed statistical correlation with mutational variants (*p* = 0.005), with the *BRAF+/TERT-* group having smaller metastatic lymph node dimensions than other mutational groups.

The *BRAF-/TERT+* mutational variant was significantly predominant in patients with distant metastases, while in patients with only locoregional involvement, *BRAF+/TERT-* status prevailed (*p* < 0.001). Most of the relevant studies showed the same results, strengthening the significance of the role that *TERTp* mutation and *BRAF/TERT* co-mutation play in the hematogenous dissemination of DTC.

Moreover, when clinical features of the tumor are observed, our results are in concordance with Blažeković et al.’s study [35], which singled out angioinvasion as a statistically significant factor (*p* = 0.02) correlated with positive *BRAF^V600E^* mutational status, as well as positive *TERTp* status in our study, which affects the dissemination stage of papillary-differentiated thyroid cancer. Most of the patients with tumor angioinvasion presented with *BRAF-/TERT+* status. However, it must be noted that the former study involved patients from all DTC risk groups, and our study only from an intermediate- and high-risk group.

There was a significant difference in age distribution between the groups; patients with *BRAF/TERT* co-mutation were significantly older at the time of diagnosis, and patients with neither of the mutations (*BRAF-/TERT-)* significantly younger (*p* < 0.001). In several previous studies, a statistically significant association between the presence of *BRAF^V600E^* and age has been described, for example, in a study conducted by Cao et al. [36].

The cumulative dose of radioiodine that intermediate- and high-risk patients received during the course of the treatment showed no significant correlation with the mutational status (*p* = 0.15), although we noticed a trend of higher cumulative radioiodine activity being applied to patients with *BRAF/TERT* co-mutation who had distant metastases. Therefore, a higher mutational burden was associated with a more unfavorable course of the disease requiring more RAI applications.

In the subgroup of patients with distant metastases, no significant association was observed between *BRAF/TERT* mutation status and the incidence of RAI-refractory disease (Table 5). Although a higher proportion of RAI-R disease was observed in patients with *BRAF/TERT* co-mutation, this did not reach statistical significance. This lack of significance may be related to limited statistical power due to the small sample size. The possibility of a Type II error cannot be excluded, and these findings should be interpreted with caution. Larger studies are needed to further evaluate the potential association between *BRAF/TERT* status and RAI refractoriness in this population.

## 5. Conclusions

The study that we conducted on intermediate- and high-risk patients with DTC showed a possible correlation between the presence of *BRAF^V600E^* and *TERTp* mutation and the effectiveness of RAI. The group of patients without *BRAF^V600E^* and *TERTp* mutation (*BRAF-/TERT-*) more often showed an excellent response to RAI, while the patient group with *BRAF/TERT* co-mutation had a predominantly incomplete or indeterminate response to RAI. In a subgroup of patients with distant metastatic disease, that correlation between the presence of *BRAF^V600E^* and *TERTp* and response to RAI was not statistically significant, but the tendency of a higher frequency of iodine -refractory disease in patients with higher metastatic burden was observed, i.e., in patients with *BRAF^V600E^* and *TERTp* co-mutation.

Furthermore, this study affirmed that patients’ *BRAF/TERT* mutational status correlated with age (patients with both mutations being significantly older), locoregional lymph node involvement, the diameter of the largest positive lymph node, angioinvasion (showing tumor aggressiveness), and the presence of distant metastases.

The presence of *BRAF/TERT* co-mutation may be associated with a less favorable disease course and poorer response to RAI. However, findings in patients with distant metastases should be considered exploratory due to the limited sample size. Larger studies are needed to further evaluate the potential association between *BRAF/TERT* status and RAI refractoriness in this population.

## 6. Limitations

This study has several limitations. First, the relatively small sample size, especially after subgroup stratification, limits the statistical power of the analysis and results in wide confidence intervals. Second, due to the limited number of events, the multivariate model was restricted to a small number of variables to avoid overfitting, which may have limited the assessment of the independent effects of all relevant clinical factors. Also, the heterogeneity of the study population, including differences in disease stage and variability in treatment protocols, is a limitation; however, it reflects real-world clinical practice in a referral center setting and may enhance the generalizability of the findings. Finally, the retrospective study design may introduce potential selection and information bias.

## Figures and Tables

**Table 1:** Association between *BRAF/TERT* mutation status and risk groups in thyroid carcinoma.

	Number of Participants, n (%)					*p* * Value
	*BRAF+* *TERT+*	*BRAF+* *TERT-*	*BRAF-* *TERT+*	*BRAF-* *TERT-*	Total	
Groups						
Intermediate and high risk	14 (17.5)	36 (45.0)	1 (1.3)	29 (36.2)	80 (100)	**<0.001**
Distant metastases	7 (23.3)	4 (13.3)	8 (26.7)	11 (36.7)	30 (100)	

* Chi-square test. Percentages are calculated within rows. Bold value denotes statistical significance.

**Table 2:** Clinicopathological characteristics according to *BRAF* and *TERT* mutation status.

	Number of Participants, n (%)					*p* * Value
	*BRAF+* *TERT+*	*BRAF+* *TERT-*	*BRAF-* *TERT+*	*BRAF-* *TERT-*	Total	
Gender						
M	14 (67)	18 (45)	4 (44)	12 (30)	48 (44)	0.06
F	7 (33)	22 (55)	5 (56)	28 (70)	62 (56)	
Metastatic status						
Locoregional involvement	14 (67)	36 (90)	1 (11)	29 (72.5)	80 (73)	**<0.001**
Distant	7 (33)	4 (10)	8 (89)	11 (27.5)	30 (27)	
T classification						
1	0	0	0	6 (16)	6 (6)	0.09
1a	0	4 (10)	1 (14)	4 (11)	9 (9)	
1b	5 (25)	14 (36)	0	8 (22)	27 (26)	
2	2 (10)	5 (13)	2 (29)	7 (19)	16 (16)	
3	11 (55)	14 (36)	4 (57)	11 (30)	40 (39)	
4	2 (10)	2 (5)	0	1 (3)	5 (5)	
N classification						
0	1 (5)	0	3 (43)	7 (19)	11 (11)	**0.01**
1	10 (50)	18 (46)	3 (43)	11 (30)	42 (41)	
1a	3 (15)	13 (33)	0	7 (19)	23 (22)	
1b	6 (30)	8 (21)	1 (14)	12 (32)	27 (26)	
M classification						
No distant meta or not confirmed at the time of operation	14 (70)	36 (92)	0	28 (76)	78 (76)	**<0.001**
Distant meta present	6 (30)	3 (8)	7 (100)	9 (24)	25 (24)	
DTC type						
Papillary	20 (95)	39 (98)	7 (78)	37 (93)	103 (94)	0.24
Follicular	1 (5)	1 (3)	2 (22)	2 (5)	6 (5)	
Mixed papillary-follicular	0	0	0	1 (3)	1 (1)	
Lobe and/or isthmus involvement						
Unifocal disease Bilateral/multifocal disease	14 (67) 7 (33)	31 (79) 8 (21)	9 (100) 0	33 (92) 3 (8)	87 (83) 18 (17)	0.05
Extrathyroidal extension (PHD)	11 (55)	13 (33)	3 (33)	13 (35)	40 (38)	0.39
Intrathyroidal dissemination (PHD)	8 (38)	18 (46)	6 (67)	16 (42)	48 (45)	0.54
Angioinvasion	3 (16)	2 (5)	4 (44)	9 (24)	18 (17)	**0.02**
Neck metastases (PHD)	17 (81)	38 (97)	3 (38)	31 (79)	89 (83)	**<0.001**
LNM with extranodal extension	1 (8)	5 (15)	0	5 (17)	11 (14)	0.94
Neck metastases at first follow-up	7 (37)	8 (21)	2 (29)	8 (22)	25 (25)	0.55
Distant metastases	7 (33)	3 (8)	8 (89)	9 (23)	27 (25)	**<0.001**

* Chi-square test. Bold values denote statistical significance. LNM: lymph node metastases.

**Table 3:** Comparison of clinical parameters according to *BRAF* and *TERT* mutation status.

	Median (IQR)				*p* * Value
	*BRAF+* *TERT+*	*BRAF+* *TERT-*	*BRAF-* *TERT+*	*BRAF-* *TERT-*	
	(1)	(2)	(3)	(4)	
Age (years)	66 (54–75)	46 (34–56)	61 (25–66)	31 (24–48)	**<0.001** ^†^
PHD—largest lesion diameter (cm)	2.0 (1.5–3.6)	1.8 (1.2–2.5)	2.5 (1.3–5.5)	2.0 (1.2–3)	0.43
LNM—largest positive lymph node diameter (cm)	1.7 (1.4–4)	0.8 (0.4–1.5)	2.5 (n = 1)	1.5 (1.1–2.7)	**0.005** ^‡^
Intermediate and high risk					
Cumulative dose of radioiodine	140 (100–201)	101 (65–127)	184 (n = 1)	100 (96–142)	0.15
Distant metastases					
Cumulative dose of radioiodine	665 (394–974)	514 (107–670)	210 (131–322)	408 (308–821)	0.12

* Kruskal–Wallis test (post hoc test Conover). Bold values denote statistical significance. Abbreviation: IQR—interquartile range. ^†^ Significant differences (*p* < 0.05) between: (1) vs. (2, 4); (2) vs. (4). ^‡^ Significant differences (*p* < 0.05) between: (2) vs. (1, 4). LNM: Lymph node metastases.

**Table 4:** Association of BRAF/TERT mutation status with therapeutic response categories (intermediate- and high-risk patients).

	Number of Participants, n (%) [95% CI]					* *p* Value
	Excellent Response (n = 35)	Incompl. Biochem. Response (n = 15)	Incompl. Struct. Response (n = 14)	Indeterminate Response (n = 2)	Total (n = 66)	
*BRAF+*	15 (51.7) [34.4–68.6]	8 (27.6) [14.7–45.7]	4 (13.8) [5.5–30.6]	2 (6.9) [1.9–22.0]	29 (44)	**0.04**
*TERT+*	0 [0.0–79.3]	0 [0.0–79.3]	1 (100) [20.7–100.0]	0 [0.0–79.3]	1 (1)	
*BRAF+TERT+*	3 (25.0) [8.9–53.2]	5 (41.7) [19.3–68.0]	4 (33.3) [13.8–60.9]	0 [0.0–24.3]	12 (18)	
*BRAF-TERT-*	17 (70.8) [50.8–85.1]	2 (8.3) [2.3–25.8]	5 (20.8) [9.2–40.5]	0 [0.0–13.8]	24 (36)	

* Fisher’s exact test. Percentages are calculated within rows. 95% confidence intervals were calculated using the Wilson method. Bold values denote statistical significance.

**Table 5:** Distribution of controlled and RAI-R disease according to *BRAF* and *TERT* genotype in patients with distant metastases.

	Number of Participants, n (%) [95% CI]			* *p* Value
	Controlled Disease (n = 17)	RAI-R Disease (n = 6)	Total (n = 23)	
*BRAF+*	2 (100) [34.2–100.0]	0 [0.0–65.8]	2 (9)	0.42
*TERT+*	7 (87.5) [52.9–97.8]	1 (12.5) [2.2–47.1]	8 (35)	
*BRAF+TERT+*	3 (50.0) [18.8–81.2]	3 (50.0) [18.8–81.2]	6 (26)	
*BRAF-TERT-*	5 (71.4) [35.9–91.8]	2 (28.6) [8.2–64.1]	7 (30)	

* Fisher’s exact test. Percentages are calculated within rows. 95% confidence intervals were calculated using the Wilson method.

**Table 6:** Multivariate logistic regression analysis of predictors of non-excellent response to RAI.

	β	OR	95% CI	*p* Value
Age (years)	−0.002	0.99	0.97–1.03	0.89
*BRAF+/TERT+* status	−1.194	0.30	0.08–1.17	0.08
Distant metastases (M1)	−1.569	0.21	0.05–0.84	**0.03**

OR: odds ratio; CI: confidence interval; β: regression coefficient (log odds). Bold values denote statistical significance.

## Data Availability

The data presented in this study are available upon request from the corresponding author.
